# Diagnostic Value of Multimodal Intraoperative Neuromonitoring by Combining Somatosensory-With Motor-Evoked Potential in Posterior Decompression Surgery for Thoracic Spinal Stenosis

**DOI:** 10.3389/fnins.2022.879435

**Published:** 2022-06-10

**Authors:** Tun Liu, Liang Yan, Huaguang Qi, Zhenguo Luo, Xuemei Liu, Tao Yuan, Buhuai Dong, Yuanting Zhao, Songchuan Zhao, Houkun Li, Zhian Liu, Xucai Wu, Fei Wang, Wentao Wang, Yunfei Huang, Gang Wang

**Affiliations:** ^1^Department of Anesthesiology, Xi’an Honghui Hospital, Xi’an Jiaotong University Health Science Center, Xi’an, China; ^2^Department of Spine Surgery, Xi’an Honghui Hospital, Xi’an Jiaotong University Health Science Center, Xi’an, China; ^3^Department of Functional Inspection Section, Xi’an Honghui Hospital, Xi’an Jiaotong University Health Science Center, Xi’an, China; ^4^The Key Laboratory of Biomedical Information Engineering, Ministry of Education, Institute of Biomedical Engineering, School of Life Sciences and Technology, Xi’an Jiaotong University, Xi’an, China; ^5^Institute of Orthopedic Surgery, Xijing Hospital, Fourth Military Medical University, Xi’an, China

**Keywords:** somatosensory-evoked potential, motor-evoked potential, postoperative neurological deficits, thoracic spinal stenosis, intraoperative neuromonitoring

## Abstract

**Background:**

Intraoperative neuromonitoring (IONM) has become an increasingly essential technique in spinal surgery. However, data on the diagnostic value of IONM in predicting impending postoperative neurological deficits (PONDs) for patients who underwent posterior decompression surgery for thoracic spinal stenosis (TSS) are limited. Furthermore, patients who are at the highest risk of waveform changes during the surgery remain unknown. Our purpose was to (1) assess the diagnostic accuracy of IONM by combining somatosensory-evoked potential (SSEP) with motor-evoked potential (MEP) in predicting PONDs for patients who underwent the surgery and (2) identify the independent risk factors correlated with IONM changes in our study population.

**Methods:**

A total of 326 consecutive patients who underwent the surgery were identified and analyzed. We collected the following data: (1) demographic and clinical data; (2) IONM data; and (3) outcome data such as details of PONDs, and recovery status (complete, partial, or no recovery) at the 12-month follow-up visit.

**Results:**

In total, 27 patients developed PONDs. However, 15, 6, and 6 patients achieved complete recovery, partial recovery, and no recovery, respectively, at the 12-month follow-up. SSEP or MEP change monitoring yielded better diagnostic efficacy in predicting PONDs as indicated by the increased sensitivity (96.30%) and area under the receiver operating characteristic (ROC) curve (AUC) value (0.91). Only one neurological deficit occurred without waveform changes. On multiple logistic regression analysis, the independent risk factors associated with waveform changes were as follows: preoperative moderate or severe neurological deficits (*p* = 0.002), operating in the upper- or middle-thoracic spinal level (*p* = 0.003), estimated blood loss (EBL) ≥ 400 ml (*p* < 0.001), duration of symptoms ≥ 3 months (*p* < 0.001), and impairment of gait (*p* = 0.001).

**Conclusion:**

Somatosensory-evoked potential or MEP change is a highly sensitive and moderately specific indicator for predicting PONDs in posterior decompression surgery for TSS. The independent risks for IONM change were as follows: operated in upper- or middle-thoracic spinal level, presented with gait impairment, had massive blood loss, moderate or severe neurological deficits preoperatively, and had a longer duration of symptoms.

**Clinical Trial Registration:**

[http://www.chictr.org.cn]; identifier [ChiCTR 200003 2155].

## Introduction

Postoperative neurological deficits (PONDs) resulting from spinal decompression surgery are the most feared complications (Thirumala P. et al., 2017; Muralidharan et al., 2020). Based on the use of different techniques to test spinal neural integrity, intraoperative neuromonitoring (IONM) can assess real-time spinal neurological function (Nuwer, 2019; Nuwer and Schrader, 2019). Multimodal IONM involves motor-evoked potential (MEP) and somatosensory-evoked potential (SSEP) (Nwachuku et al., 2015), and is a frequently used and reliable method (Ney et al., 2015). These evoked potentials (EPs) play a complementary role in detecting intraoperative spinal injuries promptly and precisely (Nuwer et al., 2012; Thirumala et al., 2016).

It was reported that the most frequent IONM waveforms change time point was during decompression in thoracic spinal surgery (Kobayashi et al., 2021). However, current IONM-relevant reports on posterior decompression surgery for the thoracic spine are limited due to the rarity of the surgery. These studies focused on SSEP (Thirumala P. D. et al., 2017; Melachuri et al., 2020), MEP (Imagama et al., 1976; Wang et al., 2017), or combined SSEPs with MEP but included relatively small sample sizes (Eggspuehler et al., 2007; Lakomkin et al., 2018). Furthermore, few investigations have explored risk factors for multimodal IONM change during posterior decompression surgery for thoracic spinal stenosis (TSS).

Our purpose was to perform a retrospective study to (1) summarize the diagnostic value of multimodal IONM by combining SSEP with MEP in posterior decompression surgery for TSS in patients with neurological deficits and (2) identify patients who were at the highest risk of IONM waveform change based on univariate and multivariate analyses. To our knowledge, this study represents the largest evaluation of the diagnostic value of combined multimodality SSEP with MEP in posterior TSS reported to date.

## Patients and Methods

### Patients

**FIGURE 1:**
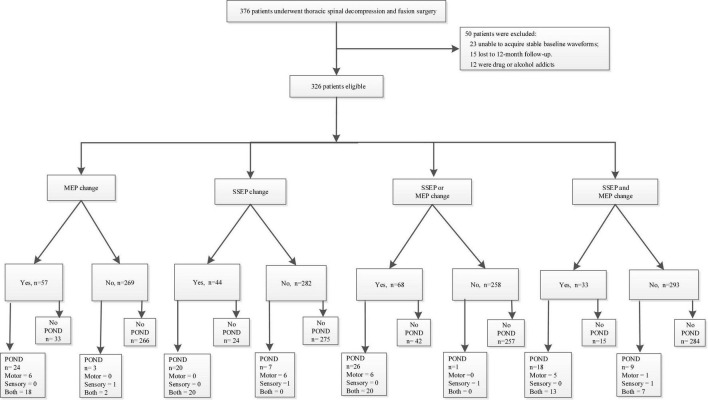
Our study flowchart. PONDs, postoperative neurological deficits; SSEP, somatosensory-evoked potential; MEP, motor-evoked potential.

**FIGURE 2:**
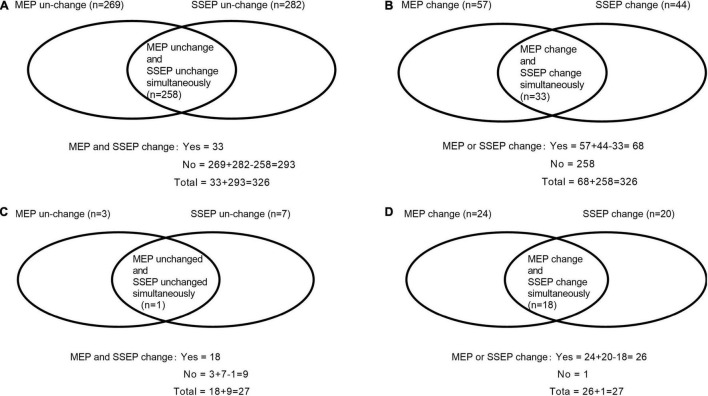
Description of the numbers of cases under single- and/or multimodal intraoperative neuromonitoring by Venn diagram, as well as the numbers of patients who developed new PONDs after posterior decompression surgery for thoracic spinal stenosis (TSS) under single- and/or multimodal intraoperative neuromonitoring. The numbers of cases with SSEP change-, MEP change-, SSEP and MEP change-, as well as SSEP or MEP change- were 44, 57, 33, and 68, respectively **(A,B)**. Furthermore, the numbers of patients developed PONDs after TSS under the corresponding neuromonitoring were 20, 24, 18, and 26, respectively **(C,D)**. SSEP, somatosensory-evoked potential; MEP, motor-evoked potential; PONDs, postoperative neurological deficits.

The study was approved by the Ethical Committee and Institutional Review Board of our hospital. It was registered at ChineseClinicalTrialRegistry.cn (ChiCTR2000032155). We retrospectively identified 376 patients who underwent posterior decompression surgery for TSS between March 2010 and March 2020 in our hospital. Inclusion criteria (Liu et al., 2021) were (1) patients with an American Society of Anesthesiologists status ranging from I to III, (2) MRI studies showed evidence of thoracic spinal compression, and (3) patients presented obvious TSS symptoms. Exclusion criteria were (1) patients were unable to acquire stable baseline SSEP and/or MEP waveforms, (2) patients lost to 12-month follow-up visit, and (3) patients were drug or alcohol abusers. A total of 50 patients were excluded, including 23 patients unable to acquire stable baseline waveforms, 12 patients who were drug or alcohol addicted, and 15 patients lost to the 12-month follow-up visit. Finally, 326 patients were analyzed, and details of PONDs were assessed. Figure 1 presents our flowchart. IONM data were identified and collected as follows: SSEP change-, MEP change-, SSEP and MEP change-, SSEP or MEP change- (Thirumala et al., 2016). A simultaneous change in both SSEP and MEP was defined as SSEP and MEP change-, and a change in either or both modalities was defined as SSEP or MEP change (Thirumala et al., 2016). Figures 2A,B depicts the numbers of patients with SSEP change-, MEP change-, SSEP and MEP change-, as well as SSEP or MEP change- (Thirumala et al., 2016). Figures 2C,D depicts the numbers of patients who developed PONDs after the surgery under SSEP change-, MEP change-, SSEP and MEP change-, as well as SSEP or MEP change-.

### Anesthesia Protocol

Anesthesia was induced and maintained according to the method described in our previous study (Liu et al., 2021). Propofol 1.5–2.0 mg kg^–1^, midazolam 0.01 mg kg^–1^, sufentanil 0.4–0.6 μg kg^–1^, and cisatracurium 0.10–0.15 mg kg^–1^ were used to complete anesthesia induction. To rule out undesirable suppressive effects brought by cisatracurium, a real-time train-of-four ratio was performed before eliciting MEP. Anesthesia maintenance was performed by the Diprifusor propofol infusion system, with a target-controlled infusion of propofol 2.0–4.0 μg ml^–1^ and remifentanil 0.15–0.30 μg kg^–1^ min^–1^, and then cisatracurium 1.5–2.5 mg kg^–1^ min^–1^. The depth of anesthesia was adjusted by varying the infusion speed of propofol or remifentanil based on bispectral monitor (BIS, Aspect Medical Systems Inc., United States), and mean atrial pressure (MAP) was maintained from 70 to 80 mmHg.

### Acquisition of Somatosensory-Evoked Potential and Motor-Evoked Potential

A team of electro-neurophysiologists who were in charge of recording IONM data are also in charge of identifying IONM waveforms change. MEP waveforms were recorded to abductor hallucis (AH) muscles in the lower extremities and the first dorsal interosseous muscles in the upper extremities (control). Previous studies demonstrated that AH muscles had the highest baseline rate, even in patients with preoperative severe motor deficit (Kobayashi et al., 1976). We placed the stimulation electrodes (Xi’an Friendship Med Electronics Co., China) over motor cortex regions C_3_–C_4_ according to the 10/20 EEG international system. We inserted recording electrodes into the AH muscles and the first dorsal interosseous muscles. The MEP parameters were as follows: constant voltage (220–360 V), multiple trains (5–8 pulses), and duration (300 μs). The bandpass filter ranged from 10 to 1,500 Hz. The time base was 100 ms window. The stimulations were delivered by an IONM apparatus (Cascade, Cadwell Laboratories Inc., United States).

Somatosensory-evoked potential waveforms were recorded to the median nerve (control) and posterior tibial nerve. We performed median nerve stimulation bilaterally at the wrist and posterior tibial nerve stimulation bilaterally at the head of the fibula or the medial malleolus of the ankle. The SSEP parameters were as follows: the stimulation in the median nerve was 15 mA, and in the posterior tibial nerves was 25 mA. Our single pulse was set from 5.0 to 5.7 Hz, and SSEP was displayed in a 100 ms window. A total of 300–400 stimulation repetitions were averaged to record each SSEP sweep.

### Criteria for Intraoperative Neuromonitoring Parameter Changes

We adopted IONM change criteria as follows (Nuwer and Schrader, 2019): (1) a change in SSEP was defined as a decrease of greater than 50% in amplitude of the baseline cortical wave, or as reported per each case and (2) a change in MEP was defined as a decrease of more than 80% in amplitude of the baseline value, or as reported per case.

After the corresponding interventions, a decrease in the SSEP amplitude of less than 50% and a decrease in the MEP amplitude of less than 80% compared with those recorded at baseline were defined as recovery waveforms.

We classified each IONM case as one of the following classifications (Thirumala et al., 2016; Melachuri et al., 2017): true positives (TPs), SSEP and/or MEP waveform change followed by PONDs; true negatives (TNs), no SSEP and/or MEP waveform change and no PONDs; false positives (FPs), SSEP and/or MEP waveform change but no PONDs; false negatives (FNs), no SSEP and/or MEP waveform change followed by PONDs.

Our previous study (Liu et al., 2021) on decompression surgery for the thoracic spine demonstrated that amplitude exhibits better prognostic value in predicting the postoperative neurological recovery rate compared with latency. Therefore, we only evaluated IONM amplitude.

### Clinical Assessment

Postoperative neurological deficits were detected by comparing the pre- and postoperative physical examination findings, including symptoms of motor deficits, sensory deficits, and both motor and sensory deficits. PONDs were assessed by the corresponding orthopedic surgeons before surgery, at discharge, and during the 3-, 6-, and 12-month follow-up visits. Furthermore, the orthopedic surgeon assessed the patients’ PONDs and categorized the patients as having complete recovery, partial recovery, or no recovery.

### Statistical Analysis

All data were analyzed using SPSS 24.0 statistics software (SPSS 24.0, Chicago, IL, United States). All measurement data and enumeration data are reported as mean ± standard deviation (X ± SD). Independent two-sample *t*-test was used to compare the differences between groups when the data were normally distributed, whereas Mann–Whitney *U*-test was performed to determine differences between two groups when the data were skewed. Chi-square testing was used. We calculated the area under the receiver operating characteristic (ROC) curve (AUC) and its 95% confidence interval (CI) to evaluate the diagnostic accuracy of waveform change. The correlation between independent risk factors and postoperative JOA recovery rate (RR) was determined using the Pearson correlation coefficient.

We performed univariate logistic regression to evaluate the relationship between different clinical factors and IONM change. If variables had a *p*-Value of < 0.2 in univariate regression analysis, it was further analyzed in multiple logistic regression (Tetreault et al., 2013). Furthermore, we performed multiple logistic regression to identify the independent risk factors associated with the waveform change.

Age, number of decompression levels involved, estimated blood loss, duration of symptoms, and impairment of gait were dichotomized for logistic regression. Preoperative myelopathy was categorized as mild (JOA score ≥ 15), moderate (JOA = 12–14), or severe (JOA < 12) (Fehlings et al., 2013). The operated thoracic spinal level was categorized as upper (T_1–4_), middle (T_5–8_), and lower (T_9–12_) (Kobayashi et al., 2021). The cutoff values of symptoms duration^21^ were consistent with previous studies. The cutoff values of other variables were deemed clinically appropriated by all authors.

## Results

### Demographic and Perioperative Data of the Study Population

Demographic and perioperative data are depicted in Table 1. In total, SSEP change-, MEP change-, SSEP and MEP change-, and SSEP or MEP change- were noted in 44, 57, 33, and 68 patients, respectively. A total of 27 patients developed PONDs, including only motor deficits (*n* = 6), only sensory deficits (*n* = 1), and both motor and sensory deficits (*n* = 20). These findings are depicted in Figures 1, 2.

**TABLE 1:** Summary of demographic and preoperative data.

	Demographic and preoperative data	Statistics
	Demographic data	Mean ± SD, Range
	Age (y)	41.23 ± 15.20, 23–70
	Sex (M/F)	170/156
*:*	Height (cm)	165.42 ± 6.52, 141–185
	Weight (kg)	62.45 ± 13.43, 41–102
*M:*	Duration of symptom (months)	3.21 ± 2.75, 0.3–23
	Operation time (min)	173.56 ± 71.35, 70–870
	Bleeding volume (ml)	302.15 ± 102.30, 100–2800
	Comorbidities	Case (n = )
	Chronic hypertension (Yes/No)	69/257
	Diabetes mellitus (Yes/No)	68/258

*M, male; F, female. Data are expressed as mean ± standard deviation (SD) and range for age, height, weight, duration of symptom, operation time, bleeding volume, and the number of the sex (male or female). OPLL, ossification of the posterior longitudinal ligament; OYL, ossification of the yellow ligament; TSS, thoracic spinal stenosis.*

### Diagnostic Value of Multimodal Intraoperative Neuromonitoring

The efficacy of IONM is presented in Table 2.

**TABLE 2:** Analysis of the efficacy of single- and multimodal intraoperative neuromonitoring waveform changes.

IONM waveform	N	TP	FP	TN	FN	Sensitivity (%)	Specificity (%)	*P*-value	AUC	OR (95% CI)	PPV (%)	NPV (%)
SSEP change-	326	20	24	275	7	74.07	91.97	<0.001	0.830	32.74 (12.58, 85.20)	45.45	97.52
MEP change-	326	24	33	266	3	88.89	88.96	<0.001	0.889	64.49 (18.41, 225.86)	42.11	98.88
SSEP or MEP change-	326	26	42	257	1	96.30	85.95	<0.001	0.911	159.10 (21.03, 1203.83)	38.24	99.61
SSEP and MEP change-	326	18	15	284	9	66.67	94.98	<0.001	0.808	37.87 (14.59, 98.29)	54.55	96.93

*TP, true positive; FP, false positive; TN, true negative; FN, false negative; AUC, area under the receiver operating characteristic curve; OR, odds ratio; PPV, positive predictive value; NPV, negative predictive value; SSEP, somatosensory evoked potential; MEP, motor evoked potential. Sensitivity = true positive/(true positive + false negative); specificity = true negative/(false positive + true negative); positive likelihood ratio = sensitivity/(1 – specificity); negative likelihood ratio = (1 – sensitivity)/specificity; positive predictive value = true positive/(true positive + false positive); negative predictive value = true negative/(true negative + false negative).*

Somatosensory-evoked potential or MEP change- yielded the following features: sensitivity 96.30%, specificity 85.95%, AUC value 0.911, odds ratio (OR) 159.10 [95% CI (21.03, 1203.83)], positive predictive value (PPV) 38.24%, and negative predictive value (NPV) 99.61%. Only one neurological deficit occurred without SSEP or MEP change.

Somatosensory-evoked potential and MEP change- showed the following features: sensitivity 66.67%, specificity 94.98%, AUC value 0.808, OR 37.87 [95% CI (14.59, 98.29)], PPV 54.55%, and NPV 96.93%. However, there were nine confirmed cases of neurological deficits that occurred without SSEP and MEP change.

### Details of Interventions, and the Number of Improved Waveform Cases After Interventions

Details of our interventions to address waveform changes intraoperatively and the number of waveforms that improved after interventions are shown in Table 3. The total numbers of cases of recovery waveforms intraoperatively in patients classified with SSEP change-, MEP change-, SSEP and MEP change-, and SSEP or MEP change- were 24, 33, 15, and 42, respectively.

**TABLE 3:** Details of our interventions and interventions lead to waveforms improvement in the corresponding groups.

	SSEP change-	MEP change-	SSEP and MEP change-	SSEP or MEP change-
**Single intervention**				
BP increased	2	4	2	4
Neuro-muscular blockers	0	2	0	2
Stimulation increased	0	2	0	2
Administration corticosteroids	2	3	1	4
Change surgical position	1	0	0	1
Surgery related manipulation	5	7	3	9
**Combined interventions**				
Stimulation increased + corticosteroids administration	3	5	3	5
BP increased + corticosteroids administration	3	2	2	3
BP increased + Surgery related manipulation	4	5	3	6
Stimulation increased + Surgery related manipulation	4	3	1	6
Total	24	33	15	42

*BP, blood pressure; MEP, motor-evoked potential; SSEP, somatosensory-evoked potential.*

### Factors Correlated With Somatosensory- Evoked Potential or Motor-Evoked Potential Waveforms Change

In univariate analysis, the following factors that were associated with a high probability of multimodal IONM waveforms change, namely, operated level in upper- or middle-thoracic spine level (*p* < 0.001), decompression in multilevel (*p* = 0.12), estimated blood loss ≥ 400 ml (*p* < 0.001), preoperative moderate or severe myelopathy (*p* = 0.03), duration of symptoms ≥ 3 months (*p* < 0.001), and impairment of gait (*p* < 0.001). In multiple logistic regression analysis, the independent risk factors were as follows: operated in upper- or middle-thoracic spine level [OR 0.53, 95% CI (0.35, 0.81), *p* = 0.003], estimated blood loss ≥ 400 ml [OR 5.12, 95% CI (2.39, 10.97), *p* < 0.001], preoperative moderate or severe myelopathy [OR 2.66, 95% CI (1.44, 4.94), *p* = 0.002], duration of symptoms ≥ 3 months [OR 5.55, 95% CI (2.42, 12.72), *p* < 0.001], and gait impairment [OR 6.79, 95% CI (2.20, 20.88), *p* = 0.001], as presented in Table 4.

**TABLE 4:** Univariate and multivariate logistic analysis of factors associated with a high probability of “SSSEP or MEP change” (*n* = 68) during posterior decompression surgery for thoracic spinal stenosis (TSS).

	No. of patients	SSEP or MEP change-		Univariate Analysis			Multivariate Analysis		
				
		Yes	No	*P*-value	OR	95% CI	*P*-value	OR	95% CI
Ages (years) (ref. = “≤50”)				0.28	0.74	(0.43, 1.27)	0.38	1.36	(0.68, 2.71)
≤50	162	30	132						
>50	164	39	125						
Sex (ref. = “Male”)				0.54	0.90	(0.53, 1.53)	0.39	1.36	(0.68, 2.76)
Male	170	35	135						
Female	156	34	122						
Operated level (ref. = “T_9_-T_12_”)				<0.001	7.23	(4.01,13.03)	**0.003**	0.53	(0.35, 0.81)
T_1_-T_4_	15	7	8						
T_5_-T_8_	94	40	54						
T_9_-T_12_	217	21	196						
Decompression level involved (ref. = “single”)				0.12	0.55	(0.26, 1.18)	0.11	2.21	(0.83, 5.85)
Single-	65	9	56			-			
Multi-	261	59	202						
Estimated blood loss (ml) (ref. = “<400 ml”)				<0.001	5.11	(2.70, 9.66)	<**0.001**	5.12	(2.39, 10.97)
<400	160	14	146						
≥400	166	54	112						
Preoperative myelopathy (ref. = “≥16”)				0.03	2.33	(1.01, 5.12)	**0.002**	2.66	(1.44, 4.94)
Mild (≥16)	74	8	66						
Moderate (12 to 14)	188	46	142						
Severe (≤12)	64	13	51						
Duration of symptoms (months) (ref. =<3 months)				<0.001	7.90	(3.76,16.61)	<**0.001**	5.55	(2.42, 12.72)
≥3	175	59	116						
<3	151	9	142						
Impairment of gait (ref. = “no”)				<0.001	9.93	(4.56,21.62)	**0.001**	6.79	(2.20, 20.88)
Yes	171	60	111						
No	155	8	147						
Smoker (ref. = “no”)				0.59	1.33	(0.63, 2.78)	0.76	1.13	(0.52, 2.46)
Yes	69	11	58						
No	257	48	209						
Chronic hypertension (ref. = “no”)				0.74	0.90	(0.48, 1.70)	0.36	1.54	(0.61, 3.91)
Yes	69	16	53						
No	257	55	202						
Diabetes mellitus (ref. = “no”)				0.42	0.76	(0.42, 1.39)	0.09	0.45	(0.18, 1.14)
Yes	68	19	49						
No	258	60	198						

*MEP, motor-evoked potential; SSEP, somatosensory-evoked potential; TSS, thoracic spine stenosis. CI indicates confidence interval. CI, confidence interval; OR, odds ratio; No. of patients, number of patients; SSEP and MEP change: a simultaneous change in both SSEP and MEP; SSEP or MEP change: a change in either or both modalities.*

### Details of Patients Who Developed New Postoperative Neurological Deficits at the 12-Month Follow-Up

In total, 27 patients developed PONDs. At the 12-month follow-up, 15, 6, and 6 patients achieved complete recovery, partial recovery, and no recovery, respectively, as depicted in Table 5.

**TABLE 5:** Details of patients developed into postoperative neurological deficits after posterior decompression for thoracic spinal stenosis.

No.	Details of high-risk factors	MEP amplitude in alerts (%)	SSEP amplitude in alerts (%)	The final waveforms, after interventions (compared with baseline value)		Neurological complications	Neurological outcome
				Final MEP amplitude (%)	Final SSEP amplitude (%)		
1	➀➁➂➃➄	Totally lost	Totally lost	0	0	Paraparesis	No recovery
2	➀➁➂➃➄	Totally lost	Totally lost	0	15	Paraparesis	No recovery
3	➀➁➂➃➄	Totally lost	Totally lost	0	0	Paraparesis	No recovery
4	➀➁➂➃➄	Totally lost	Totally lost	0	28	Paraparesis	No recovery
5	➀➁➂➃➄	Totally lost	Totally lost	0	0	Deterioration of pre-existing paraparesis, as well as partial bladder incontinence.	No recovery
6	➀➁➂➃➄	Totally lost	Totally lost	0	23	Deterioration of pre-existing paraparesis.	No recovery
7	➀➁➂➄	Totally lost	28	10	59	Deterioration of pre-existing mild partial sensory-motor deficits in LE.	Partial recovery
8	➀➁➃➄	Totally lost	40	0	67	Deterioration of pre-existing weakness and mild motor deficits in LE, as well as partial bladder incontinence.	Partial recovery
9	➀➁➃➄	Totally lost	38	0	61	Deterioration of pre-existing mild partial sensory-motor deficits in LE.	Partial recovery
10	➀➁➂➄	Totally lost	31	10	72	Deterioration of pre-existing weakness and mild motor deficits in LE.	Partial recovery
11	➀➁➂	10	25	10	68	Deterioration of pre-existing weakness and mild motor deficits in LE.	Partial recovery
12	➀➁➂	Totally lost	42	15	70	Mild partial sensory-motor deficits in left LE.	Partial recovery
13	➁➂➃	10	65	50	81	Deterioration of pre-existing LE weakness.	Complete recovery at discharge
14	➂➃	10	68	50	65	Mild partial sensory-motor deficits in LE	Complete recovery at discharge
15	➁➂➃	10	73	40	67	Mild partial sensory-motor deficits in LE	Complete recovery at discharge
16	➁➂➃➄	15	70	50	81	Mild partial sensory-motor deficits in LE	Complete recovery at discharge
17	➁➂➃➄	10	28	10	60	Mild partial sensory-motor deficits in LE	Complete recovery at discharge
18	➁➂➃	Totally lost	20	0	65	Mild partial sensory-motor deficits in LE	Complete recovery at discharge
19	➀➂➃	Totally lost	65	10	82	Mild partial sensory-motor deficits in LE.	Complete recovery at discharge
20	➀➁➂➃	Totally lost	32	20	65	Mild partial sensory-motor deficits in LE.	Complete recovery in 3 mons
21	➀➁➂➃	35	33	40	67	Mild partial sensory-motor deficits in LE.	Complete recovery in 3 mons
22	➀➁➂➃	15	34	43	63	Deterioration of pre-existing weakness and motor deficits in LE.	Complete recovery in 3 mons
23	➁➂➃➄	10	23	40	72	Weakness and mild motor deficits in LE.	Complete recovery in 6 mons
24	➂➃➄	Totally lost	Totally lost	20	55	Partial paraparesis	Complete recovery in 6 mons
25	➀➂➃➄	Totally lost	Totally lost	0	63	Partial paraparesis	Complete recovery in 6 mons
26	➀➁➃	25	10	40	60	Mild partial sensory-motor deficits in LE	Complete recovery in 6 mons
27	➁➂➃	No changes	No changes	55	65	Mild sensory deficits in left LE.	Complete recovery at discharge

*M, male; F, female; BL, baseline; MEP, motor-evoked potential; SSEP, somatosensory-evoked potential; TSS, thoracic spine stenosis; OPLL, ossification of yellow ligament; ISCT, intramedullary spinal cord tumors; ESCT, extramedullary spinal cord tumors; LE, lower extremity. ➀➁➂➃➄ in the table corresponded to gait impairment, operated level in upper- or middle-thoracic spine level, estimated blood loss more than 400 ml, duration of symptoms more than 3 months, and preoperative moderate or severe neurological deficits.*

Six patients developed paraparesis or deterioration of preexisting paraparesis postoperative and showed no recovery at the 12-month follow-up. Furthermore, combined SSEP with MEP waveforms were lost intraoperatively, and the final waveforms showed no recovery in those patients. All the patients had those five high-risk factors.

Six patients presented deterioration of preexisting deficits and got partial recovery. Combined SSEP with MEP waveforms changed intraoperatively. However, only the final SSEP waveforms improved after interventions. The six patients had three or four high-risk factors.

A total of 15 patients presented mild or moderate partial deficits postoperative and got complete recovery at discharge or within a 6-month follow-up. Combined waveforms changed intraoperatively in seven patients, only MEP changed in five patients, and only SSEP changed in three patients. After interventions, 11 patients presented improved waveforms, and only 4 patients presented unimproved final MEP waveforms.

Only one patient presented no changes in waveforms intraoperative but presented with mild sensory deficits in the left lower extremity postoperatively. However, the final amplitude of MEP and SSEP decreased by 55 and 65%, respectively.

## Discussion

In this study, 27 patients developed PONDs after surgery, including only motor deficits (*n* = 6), only sensory deficits (*n* = 1), and both motor and sensory deficits (*n* = 20). After 12 months of follow-up, 15 patients completely recovered, 6 patients partially recovered, and 6 patients showed no recovery. We demonstrated that SSEP or MEP change monitoring is a highly sensitive and moderately specific indicator for predicting PONDs in posterior decompression surgery for TSS. We further identified five independent risk factors associated with IONM waveforms changes in our study population as follows: operated in upper- or middle-thoracic spine level, massive blood loss, preoperative moderate or severe myelopathy, a longer duration of symptoms, and gait impairment preoperative.

Compared with SSEP and MEP change- monitoring, SSEP or MEP change- monitoring yielded better diagnostic efficacy. There are two factors for this phenomenon. (1) The blood supply is much less at the thoracic level because of the much smaller diameter in the thoracic cord (Imagama et al., 1976; Stokes et al., 2019). So, it can result in difficulties in surgical procedures and vulnerability in blood loss, even resulting in decreased spinal cord blood flow (SCBF). Previous animal studies (MacDonald et al., 2003) and clinical studies (Hilibrand et al., 2004; Schwartz et al., 2007) demonstrated MEP was more sensitive to decreased SCBF and injury, and changes in SSEP lagged behind changes in MEP by 5 (Schwartz et al., 2007) to 33 (Hilibrand et al., 2004) min. Therefore, it results in an unsimultaneous change in waveforms. (2) Furthermore, either or both of the waveforms changed, the surgical procedures were halted transiently, and corresponding interventions were immediately taken to address waveform changes.

Furthermore, orthopedic surgeons, anesthesiologists, and neurophysiologists should pay special attention to a simultaneous change in both SSEP and MEP. In patients who developed postoperative paraparesis, all of them exhibited the change (Table 5). Simultaneous SSEP and MEP changes are very specific and hazardous indicators. This finding indicates a high prognostic value for postoperative neurological recovery, which is consistent with the findings of our previous study^15^. When SSEP and MEP change simultaneously, a spinal cord injury (SCI) will likely occur, and real-time waveform changes can be observed.

We observed 26 TPs (8.00%), 42 FPs (12.89%), 257 TNs (78.81%), and 1 FNs (0.31%) in SSEP or MEP change- monitoring. Our increased number of FPs (*n* = 42) and low PPV (38.24%) can be attributed to the following factors: (1) the number of changed waveforms be improved after interventions up to 42 cases (Table 3) and (2) patients with waveform changes but without PONDs were classified into FPs rather than TPs. This results in a higher FPs rate and a low PPVs value.

Previous studies demonstrated that older age was an independent risk factor associated with IONM changes (Ghadirpour et al., 2018) or could predict surgical outcomes (Tetreault et al., 2015). However, no association between older age and waveform change was found in our study based on univariate and multivariate analysis. Furthermore, no significance was found between age-related comorbidities such as chronic hypertension or diabetes mellitus and a high probability of waveforms change. Theoretically, a gradual decrease in the number of motor neurons and anterior horn neurons can be found in elderly patients, and they are more likely to have comorbidities that may influence SCBF, and eventually influence IONM (Hasegawa et al., 2002). However, the mean age in our study was nearly 15 years younger than Fehling’s or Ghadirpour’s study population (Tetreault et al., 2015; Ghadirpour et al., 2018) (41.23 years vs. 56.48 years, and 41.23 years vs. 55.8 years, respectively). Furthermore, the duration of age-related comorbidities (such as chronic hypertension or diabetes mellitus) is relatively short. So, age-related factors are not associated with waveforms change or neurological function improvement.

We also demonstrated that patients who presented with gait impairment, had lower JOA score preoperative, and had a longer symptoms duration were also independent high risks associated with waveform change, which is consistent with previous studies (Fehlings et al., 2013; Tetreault et al., 2015). We further demonstrated patients who had massive EBL are an independent risk factor. A total of 51 patients in our study experienced massive bleeding in a short time (>500 ml of blood loss in <20 min), leading to fluctuations in cardiovascular stability and MAP. Previous studies revealed that low MAP could influence human autoregulation by maintaining stable cerebral blood flow (CBF) and SBF (Crystal et al., 2014; Meng et al., 2019). Increasing MAP alone or combined with other interventions restored 31.0% (13/42) of changed waveforms (Table 3). Therefore, we monitored arterial blood gas routinely and continually, especially during massive bleeding periods. Timely measures could be taken to address hypovolemia and/or low hemoglobin to avoid adverse effects on IONM recordings.

One case of POND occurred without waveform changes (FN), and FNs indicate that the signals cannot detect spinal injuries intraoperatively. Although the patient had no waveforms change, a decrease of 45% in MEP amplitude and a decrease of 35% in SSEP amplitude occurred. Finally, the patient presented mild sensory deficits in the left lower extremity and got complete recovery at discharge. This indicates that attention should be paid to the patients presenting with a decrease in amplitude in both SSEP and MEP, even when the decrease in amplitude did not meet the change criterion. Our future study will focus on this point.

**FIGURE 3:**
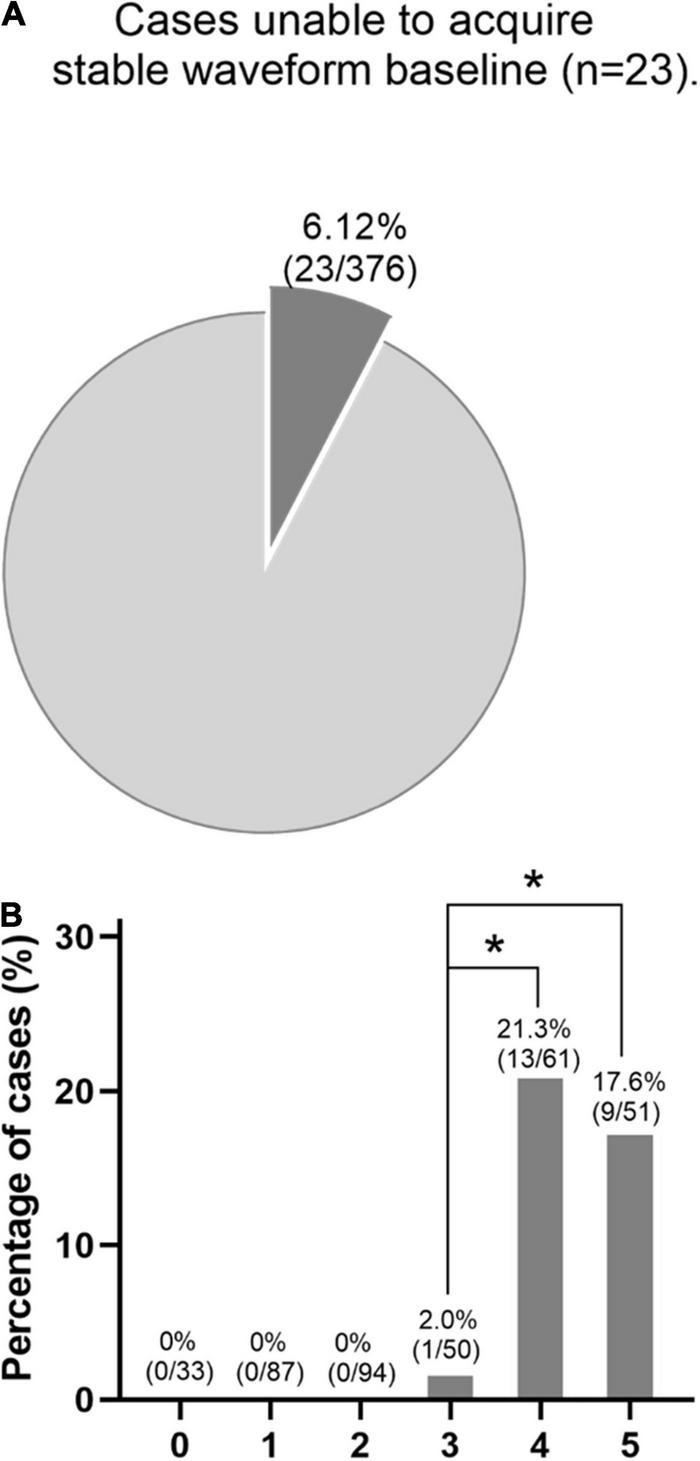
**(A)** Cases that were unable to acquire stable waveform baseline included 23 patients (6.12%). **(B)** In this series, the baseline waveforms can be detectable in patients with less than three risk factors. However, the incidence of undetectable baseline waveforms was significantly higher in patients with four or five risk factors compared with patients with three risk factors. *p < 0.05.

Cases unable to acquire stable waveform baseline included 23 patients (6.12%, 23/376), as depicted in Figure 3. The incidence of undetectable baseline waveforms was significantly higher in patients with four or five risk factors compared with patients with three factors. Furthermore, the waveforms can be detectable in patients with less than three risk factors (Figure 3B). This indicates the number of independent risk factors can exert an influence on the feasibility of a detectable waveform baseline.

### Limitations of the Study

There were several limitations to our study. First, the study was retrospective and single center, which can introduce bias in the results. It would be more convincing that data come from a prospective and multicenter study. Second, our follow-up visit was relatively short for some patients’ neurological function improvement, and a longer follow-up period is needed in the future study. Third, there was no comparison group of patients who underwent TSS without IONM. In the future study, we will schedule to perform a multicenter study focused on patients who underwent TSS surgery but without IONM guidance. Fourth, for patients who cannot acquire a stable IONM baseline (*n* = 23), we performed continuous EMG monitoring. In the future, we will introduce more complex electrophysiological monitoring, for example, D-wave monitoring. We only evaluated IONM amplitude for the following three reasons: (1) this study relied on a retrospective database from our hospital between March 2010 and March 2020; the most well-recorded data is the IONM amplitude. In the future, we will perform a prospective, randomized study that will include the latency and the morphology of the waveforms. (2) Our previous study demonstrated that amplitude exhibits better prognostic value in predicting PONDs compared with latency (Liu et al., 2021). (3) IONM waveforms latency and morphology showed a weaker sensitivity and specificity in predicting PONDs compared with the amplitude (Hilibrand et al., 2004; Nuwer and Schrader, 2019). Furthermore, MEP latency prolongation is not adopted as a change criterion in the current guidance and our hospital. So, we only chose amplitude as the IONM waveform change criteria.

## Conclusion

Somatosensory-evoked potential or MEP change is a highly sensitive and moderately specific indicator for predicting PONDs in posterior decompression surgery for TSS. It exhibits increased diagnostic accuracy. We identified five independent risks for IONM change as follows: operated in upper- or middle-thoracic spinal level, presented with gait impairment, had massive blood loss, moderate or severe neurological deficits preoperatively, and had a longer duration of symptoms.

## Data Availability Statement

The original contributions presented in the study are included in the article/Supplementary Material, further inquiries can be directed to the corresponding author/s.

## Ethics Statement

The studies involving human participants were reviewed and approved by Biomedical Research Ethics Committee, Xi’an Honghui Hospital, Xi’an Jiaotong University Health Science Center. The patients/participants provided their written informed consent to participate in this study.

## Author Contributions

TL, LY, HQ, ZLu, XL, TY, BD, YZ, SZ, HL, ZLi, XW, FW, WW, YH, and GW contributed to the design and initiation of the study, patient recruitment, monitoring of processes, compilation of the CRF database, statistical analyses, composition of the first draft of the manuscript, and preparation of the figures. GW, TY, and XL contributed to the interpretation of the data. All authors revised and approved the final version of the manuscript.

## Conflict of Interest

The authors declare that the research was conducted in the absence of any commercial or financial relationships that could be construed as a potential conflict of interest.

## Publisher’s Note

All claims expressed in this article are solely those of the authors and do not necessarily represent those of their affiliated organizations, or those of the publisher, the editors and the reviewers. Any product that may be evaluated in this article, or claim that may be made by its manufacturer, is not guaranteed or endorsed by the publisher.
